# Supplementary material for: Somatic Reversion of a Novel IL2RG Mutation Resulting in Atypical X-Linked Combined Immunodeficiency
Source: Genes (Basel). 2021 Dec 23;13(1):35. doi: 10.3390/genes13010035 (PMC8774591; doi:10.3390/genes13010035)
Supplement: Supplementary file 1 [file genes-13-00035-s001.zip › genes-1494795-supplementary.pdf]

Table S1. Sequence of Primers and Probes.

Item	Name	Sequence (5' →3')
IL2RG	For primer	AGGCCACACAGATGCTAAAACT
	Rev primer	TGCTACATTCACGTCCCTAGT
TREC	For primer	CACATCCCTTTCAACCATGCT
	Rev primer	GCCAGCTGCAGGGTTTAGG
	Probe	/6-FAM/ ACACCTCTG/ZEN/GTTTTTGTAAAGGTGCCCACT/IABkFQ/
KREC	For primer	TCCCTTAGTGGCATTATTTGATCACT
	Rev primer	AGGAGCCAGCTCTTACCCTAGAGT
	Probe	/6-FAM/TCTGCACGG/ZEN/GCAGCAGGTTGG/IABkFQ/
RPP30	For primer	AGATTTGGACCTGCGAGCG
	Rev primer	GAGCGGCTGTCTCCACAAGT
	Probe	/HEX TTCTGACCT/ZEN/GAAGGCTCTGCGCG /IABkFQ/
STR analysis	For primer	CAGGATATTATGTGATGGAATCC-6FAM
	Rev primer	GATCTCTTCTCTCTCTCTCTTTCTCCC

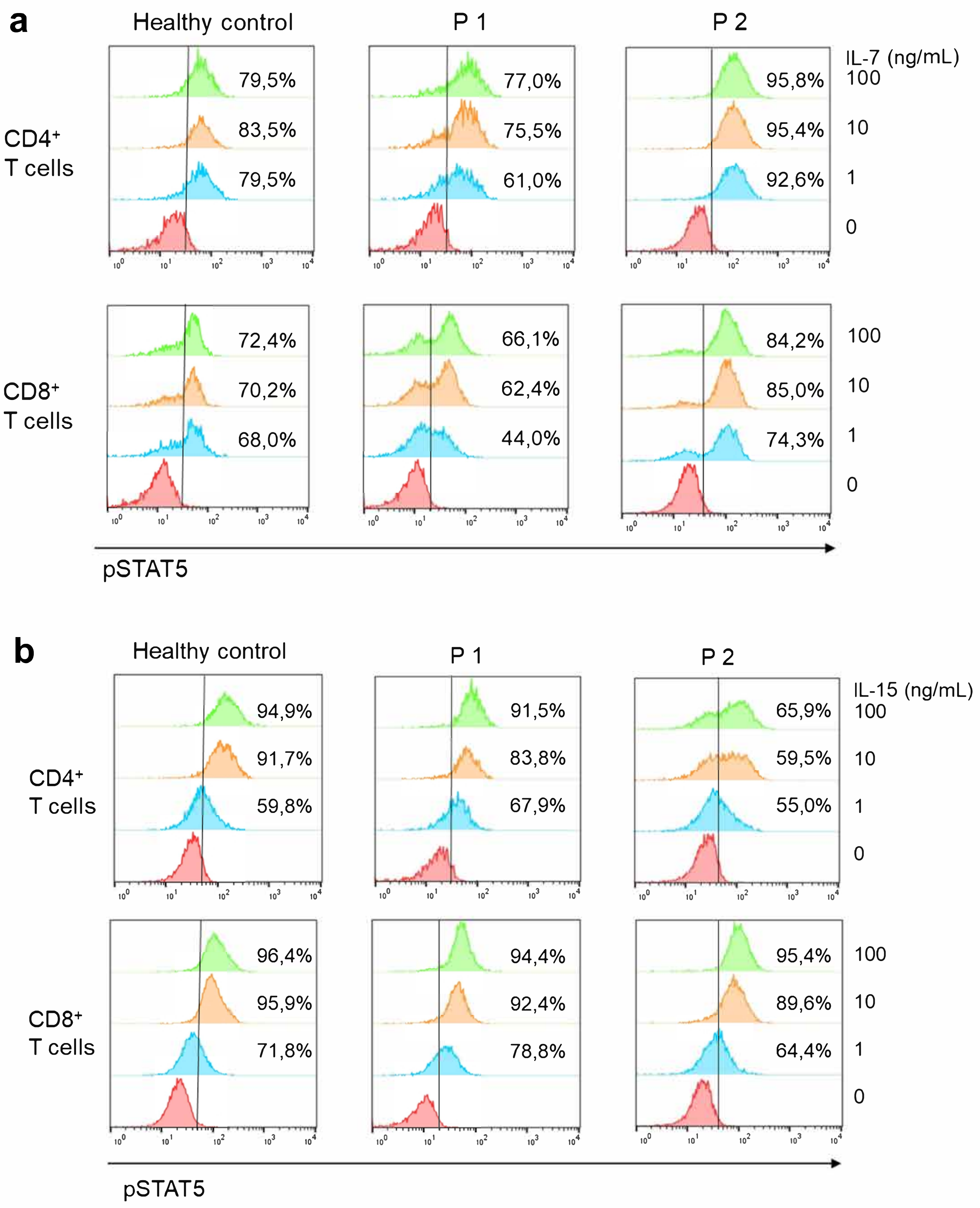

Figure S1. STAT5 phosphorylation with (a) IL-7 and (b) IL-15 using 1, 10, and 100 ng/mL. No sig-nificant difference could be observed in STAT5 phosphorylation from patient cells compared to an age-matched healthy control.

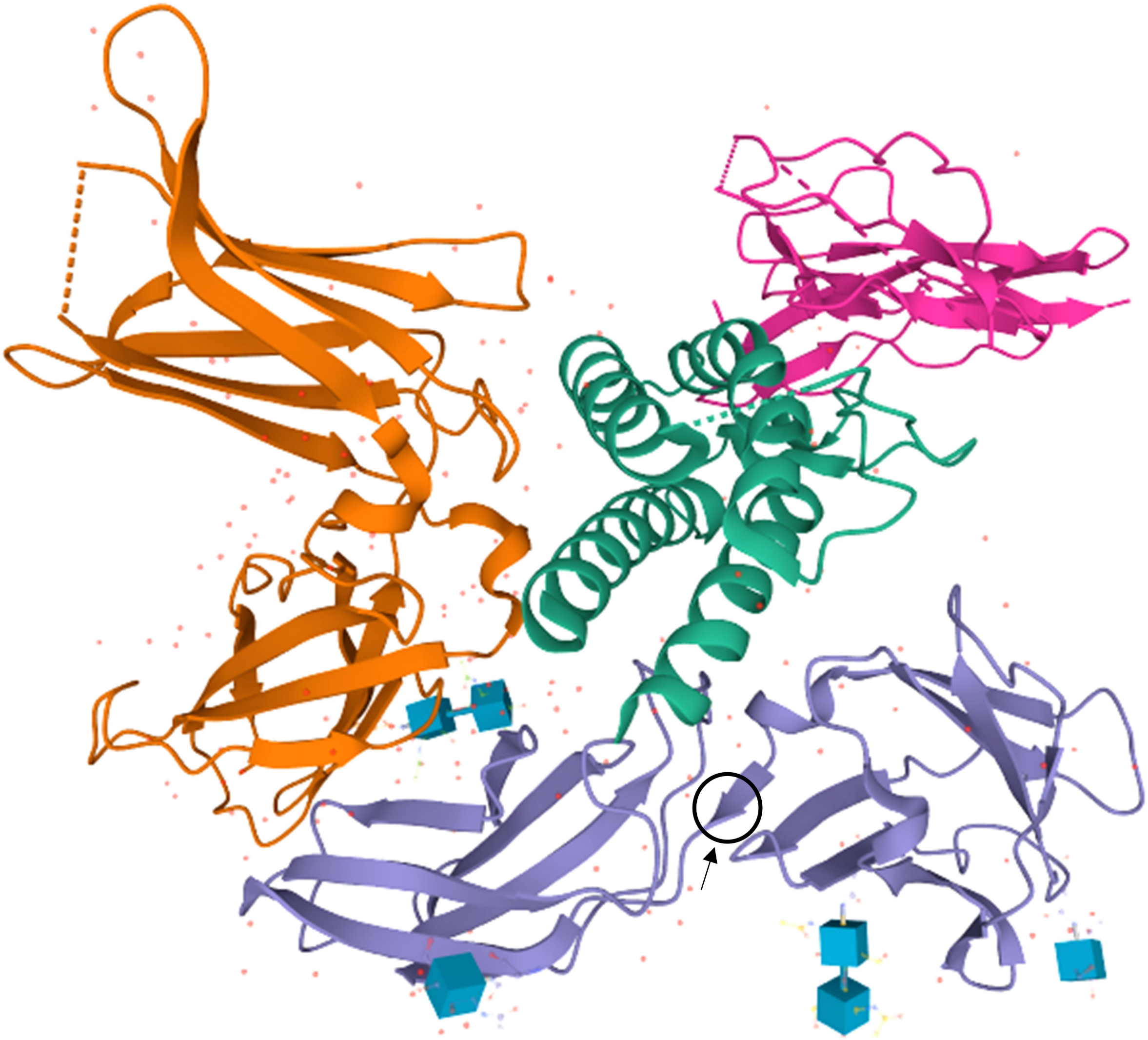

Figure S2. 3D visualization of the molecular interaction of IL-2 binding to IL-2 receptor. IL-2 is depicted in green, IL2RA in blue, IL-2RB in orange and IL-2RG in purple. Isoleucine 153 from IL-2RG (included in a β -sheet motif) is highlighted. The image was generated with Mol* Viewer application [38] in RCSB PDB (PDB ID: 2B5I [27]). The structure was analyzed by X-ray diffraction (2.30 Å of resolution) by Wang et al. [27].
